# The Effect of Hearing Aid Amplification on Gait Parameters: A Pilot Study Using Ear-Worn Motion Sensors

**DOI:** 10.3390/audiolres15030045

**Published:** 2025-04-23

**Authors:** Ann-Kristin Seifer, Arne Küderle, Kaja Strobel, Ronny Hannemann, Björn M. Eskofier

**Affiliations:** 1 Machine Learning and Data Analytics Lab (MaD Lab), Department Artificial Intelligence in Biomedical Engineering (AIBE), Friedrich-Alexander-Universität Erlangen-Nürnberg (FAU), 91052 Erlangen, Germany; 2 WS Audiology, 91058 Erlangen, Germany; 3 Translational Digital Health Group, Institute of AI for Health, Helmholtz Zentrum München—German Research Center for Environmental Health, 85764 Neuherberg, Germany

**Keywords:** earables, hearing aid, gait, accelerometer, dual tasks, amplification, gait analysis, hearing loss

## Abstract

**Background/Objectives**: Hearing loss, particularly in older adults, is associated with reduced physical functioning; increased fall risk; and altered gait patterns, including slower walking speed and shorter step length. While the underlying mechanisms are not fully understood, one possibility is that these gait changes result from an additional cognitive load due to hearing difficulties. Prior research suggests that hearing aids may improve balance; however, their impact on gait remains less well explored. **Methods**: This study investigated gait parameters in individuals with hearing loss as they walked with and without hearing aid amplification under different dual-task conditions. Additionally, we showed the potential of ear-worn sensors for detecting relevant gait changes. To achieve this, we used a hearing-aid-integrated accelerometer and our open-source EarGait framework comprising gait-related algorithms specifically developed for ear-worn sensors. **Results**: Our findings revealed no significant differences in gait velocity or step length between the unaided and aided conditions. For stride time, we observed a significant interaction effect; however, the effect size was negligible. The dual-task costs were lower than in previous reports, indicating that the applied dual-task paradigm did not induce the expected cognitive demand. The ear-worn gait analysis system showed strong performance compared to foot-worn sensors. **Conclusions**: Our findings indicate that in controlled, low-cognitive-demand settings, hearing aid amplification does not affect gait performance and, therefore, neither hinders nor improves walking performance. Additionally, the high accuracy of the ear-worn gait analysis system highlights the strong potential of ear-mounted wearable devices (“earables”) for real-world mobility assessments. Future research should explore more complex real-world conditions to better understand the impact of hearing aids on walking behavior. Our proposed earable-based system offers a promising tool for continuous, unobtrusive gait monitoring in everyday environments.

## 1. Introduction

Disabling hearing loss (HL) affects over 5% of the global population, and the prevalence is projected to rise to 10% by 2050 [1]. Beyond auditory impairment itself, HL may come with many challenges, including social isolation and depression, lower quality of life, and accelerated cognitive decline [2,3]. Notably, HL has also been linked to increased fall risks and deficits in physical health, particularly affecting balance and gait [4,5,6,7]. Individuals with hearing disabilities often exhibit reductions in walking speed [8,9,10], step length [11], and walking endurance [6], with greater declines in gait parameters observed as the severity of hearing loss increases [11]. Moreover, individuals with hearing impairments have not only poorer physical performance but also a faster decline over time [6].

Despite these well-documented gait alterations in individuals with HL, the underlying mechanisms that link diminished postural control with hearing loss seem to be numerous and are not completely understood [5]. One possible explanation rests on physiological and pathological mechanisms [4]. Age-related neural degeneration may contribute to simultaneous declines in hearing and walking abilities, while inner-ear pathologies affecting both the cochlear and vestibular systems could lead to concurrent hearing deficits and postural instability. However, studies that account for these comorbidities have not provided conclusive evidence to support this assumption [4]. Alternatively, cognitive mechanisms have been suggested as a potential link between HL and impaired mobility. Individuals with HL may allocate more cognitive resources to auditory processing, leading to reduced resources for mobility [4,12]. Given that attentional control declines with age, the inability to hear may further increase the demand for attentional resources [13]. Engaging in the simultaneous tasks of listening, processing auditory information, and walking may significantly limit overall physical performance. However, the additional listening effort while moving has not been carefully considered regarding mobility-related outcomes [14].

Previous research connecting HL and gait has largely focused on investigating inter-group gait differences between individuals with hearing deficits and those with normal hearing [10,15]; while some studies also analyzed whether HA usage affects physical performance over time, findings remain inconclusive. Chen et al. [9] reported no significant difference between HA users and non-users, whereas Martinez-Amezcua et al. [16] found that HA users exhibit better walking endurance in the long term. Although hearing interventions have shown potential benefits for balance [17,18], their direct influence on walking behavior remains unclear. To the best of our knowledge, the direct effect of HA amplification on gait patterns in real-world and cognitively demanding conditions has not been investigated yet.

Beyond their role in auditory perception, modern HAs are increasingly equipped with integrated motion sensors, presenting an opportunity to assess mobility alongside their primary function. Inertial sensors, including accelerometers, have been widely applied in mobility and gait assessments, offering valuable insights into gait patterns and impairments. Ear-worn sensors, in particular, provide distinct advantages due to their lightweight, unobtrusive design and seamless integration into daily life [19]. Recent advancements have led to the development of various algorithms for ear-based gait analysis [20,21,22,23], demonstrating promising results, though their accuracy remains slightly lower than that of foot-worn sensors [23]; while these algorithms have been technically validated and limitations are known, it has not yet been investigated whether ear-worn sensors can reliably detect meaningful changes in gait, such as those associated with HL, or serve as a viable tool for mobility monitoring under real-world conditions.

**Figure 1:**
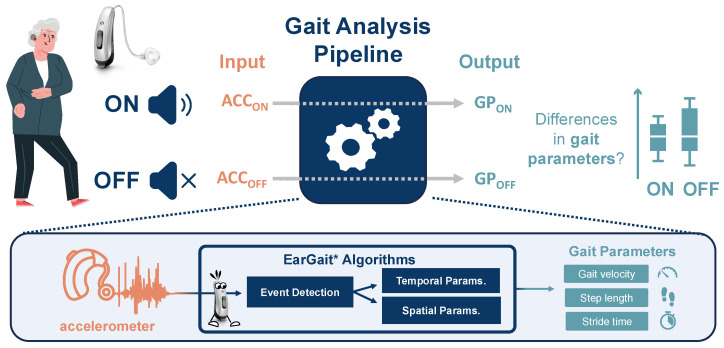
Overview of the gait analysis study investigating the effect of hearing amplification on gait parameters in dual-task scenarios. Gait parameters (GPs) were estimated using EarGait, an open-source gait analysis pipeline specifically designed for ear-worn accelerometers. ACC_ON_ and ACC_OFF_ refer to the accelerometer data recorded with hearing aid amplification enabled and disabled, respectively. The same applies to GP_ON_ and GP_OFF_, representing the corresponding gait parameters. Person illustration adapted from [24]. * https://github.com/mad-lab-fau/eargait (accessed 5 September 2024).

In this work, we investigate the effect of HA amplification on gait parameters in individuals with hearing disabilities while walking with and without HA amplification. To simulate real-world cognitive demands, we employ a dual-task paradigm that requires participants to engage in both auditory and motor tasks simultaneously. We hypothesize that HA amplification reduces cognitive load associated with auditory perception, thereby facilitating a more stable gait pattern in dual-task conditions. Furthermore, we assess the potential of a hearing aid-integrated accelerometer to measure relevant changes in gait. An open-source gait analysis toolbox that has been developed specifically for ear-worn sensors will be applied to estimate gait features; while the individual algorithms have been technically validated [22,23] and limitations have been shown, the pipeline has not yet been applied for analyzing specific gait-related research questions. Therefore, all experiments are conducted with a hearing-aid integrated accelerometer and two foot-worn inertial sensors. Figure 1 depicts a graphical summary of our work.

By investigating the influence of HA amplification on gait and evaluating the potential of ear-worn sensors for mobility assessment, this study aims to advance our understanding of the relationship between auditory function and locomotion. Our findings may contribute to the development of novel, unobtrusive monitoring solutions for individuals with HL, ultimately improving mobility assessments and fall prevention strategies in real-world environments.

## 2. Data Collection

### 2.1. Participants

**Figure 2:**
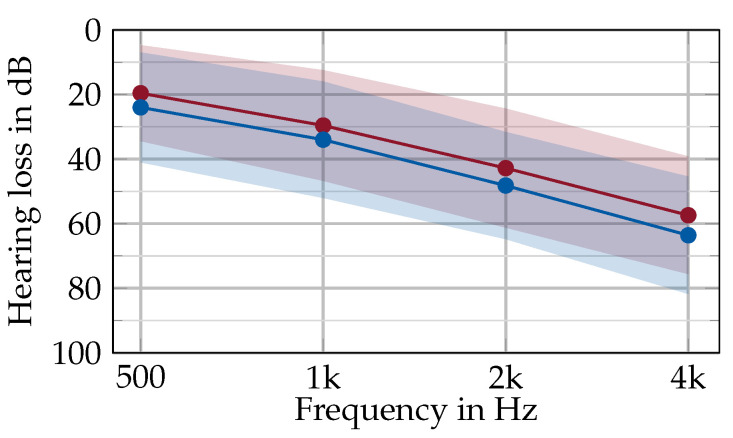
Average hearing loss across all participants for the left (blue) and right (red) ear for the main speech-specific frequencies. The shaded area depicts the standard deviation across all participants. The PTA4 (pure tone average) is 39.9 ± 11.3 dB.

A study was conducted to collect walking data for individuals with HL while walking aided and unaided. A total of 25 subjects with hearing disabilities participated in this study, and the demographic characteristics are displayed in Table 1. The participants gave written consent prior to the recording, and the study was granted ethical approval by the local ethics committee (Friedrich-Alexander-Universität Erlangen-Nürnberg, Germany; Re-No. 22-335-Sm). All participants suffered from mild to severe and bilateral hearing loss and were experienced users of hearing aids. A pure-tone audiogram was recorded by a trained professional if no recent audiogram (six months or less) was available. The PTA4 (pure tone average for four frequencies: 0.5, 1, 2, and 4 kHz) was 39.9 ± 11.3 dB and the average audiogram is depicted in Figure 2. To assess cognitive function and mobility, several standardized clinical instruments and questionnaires were applied: the Montreal Cognitive Assessment (MoCA) [25], the Fall Efficacy Scale (FES-I) [26], the Timed Up-and-Go test (TUG) [27], and the Short Physical Performance Battery (SPPB) [28]. The results of these tests are displayed in Table 1. Ten participants showed a mild cognitive impairment in the MoCA, but none of them had dementia. The SPPB and TUG assessments indicated that five participants exhibited mild mobility impairments, though these impairments remained within a range classified as having no significant impact on daily functioning.

### 2.2. Hearing Aid Fitting

Participants were equipped with a pair of hearing aids (left and right side; receiver in the canal) provided by the hearing-aid manufacturing company Sivantos GmbH. HAs were fitted with the NAL-NL2 [29] fitting prescription (experience level, adults, 100% acclimatization, vented sleeves—instant open fit). We configured two programs: one with amplification enabled and the other with amplification fully disabled, referred to hereafter as ON and OFF conditions, respectively. The OFF program was required for the unaided condition, as the motion data was recorded via the hearing aids’ integrated inertial measurement unit (IMU). Hence, participants needed to wear hearing aids for all conditions.

### 2.3. Inertial Sensors

The right HA featured an integrated IMU sensor (tri-axial accelerometer ±2 g; tri-axial gyroscope ±per-mode=symbol 1000 ^∘^/s). Motion data was collected at $f_{s}\;=\;$ 50 Hz via a smartphone app. Additionally, two foot-worn IMUs (Portabiles GmbH, Erlangen, Germany; 3D accelerometer ± 16 g; 3D gyroscope $2000^{\circ }/s$, $f_{s}=204.8\;\mathrm{Hz}$) were attached to the participant’s shoe, serving as an additional measurement system.

### 2.4. Recording Procedure

After fitting the HAs to the individual’s needs, the Freiburger speech intelligibility test was conducted in a controlled laboratory environment. The Freiburger test is a German monosyllabic word test and has been used as the gold standard for assessing word recognition scores in Germany [30]. The test was conducted while sitting in a quiet room in the ON and OFF program serving as baseline measurement (*Base_Speech_*).

Afterwards, different walking tasks were performed. For each task, participants walked along a 35 m path forth and back, which is referred to as a walking bout in the remainder of this work. To get used to the recording environment and sound amplification, a training trial was performed in which the participant walked the walking path in the ON program. To simulate real-world complexity, we used dual tasks. The dual-task paradigm has been widely applied in laboratory research to mimic real-world conditions and assess the interplay of cognitive load on specific physical or mental performance [31]. A baseline walking task was recorded along with two different dual tasks, an arithmetic and a listening dual task, resulting in the following tasks:

- *Base_Speech_*: single-task listening—performing the Freiburger monosyllabic word recognition test while sitting in a quiet environment;
- *Base_Walk_*: single-task walking;
- *DT_Calc_*: arithmetic dual task—continuously subtract 3 starting from 100 while walking;
- *DT_Listen_*: listening dual task—performing the Freiburger monosyllabic word recognition test while walking.

The three walking conditions (*Base_Walk_*, *DT_Calc_*, and *DT_Listen_*) were grouped into a recording block. The recording block was conducted twice in a randomized order, once in the OFF and once in the ON program, resulting in a total of six distinct conditions. For the Freiburger test, a sound box calibrated to 65 dB at the participant’s ear was attached to a backpack worn by the participant, and a study conductor accompanied the participant to count the number of correctly identified monosyllabic words. Between each condition, the participant was allowed to take a short break.

**Table 1:** Participants’ demographics characteristics, pure-tune average (PTA4) values, and results of cognitive and mobility assessments.

Characteristic	Mean ± Std		
Total	25		
Gender (m/f)	20% female		
Age (years)	68.2 ± 16.0		
Height (cm)	176.5 ± 7.7		
Weight (kg)	78.2 ± 13.1		
PTA4 (dB)	39.9 ± 11.3		
**Assessments**	**Mean** ± **Std**	**[Min, Max]**	**Category**
TUG (s)	9.0 ± 1.2	[6.9, 11.7]	0 fall risk [32]
MoCA	26.0 ± 2.1	[22, 30]	15 no impairment 10 MCI * [33]
FES-I	18.7 ± 2.7	[16, 26]	24 no fall risk 1 fall risk [34]
SPPB	10.5 ± 1.23	[8, 12]	20 no DIS 5 mild DIS ** [28]

PTA4—pure-tone audiometry hearing loss (0.5–4 kHz); MCI—mild cognitive impairment; DIS— disabilities; * but no dementia; ** but no impact on daily life

## 3. Methods

### 3.1. Gait Parameter Estimation

We concentrated on the estimation of three gait parameters: gait velocity, step length, and stride time, because these gait parameters are mostly reported for hearing loss-related gait changes. As described in the previous section, two sensor systems were used to record IMU data: an ear-worn system and a foot-worn system. Gait analysis algorithms are often tailored to the specific sensor positions, and both pipelines will be described in the following.

#### 3.1.1. Ear-Worn Sensors

The Python package *EarGait* [35] was used to process sensor data collected by the HA-integrated accelerometer. *EarGait* is an open-source code library designed specifically for ear-worn sensors, providing a set of algorithms and functions for processing acceleration data and extracting gait parameters. The gait analysis pipeline consists of several sequential steps. First, gait segments within each recording were identified through manual inspection of the raw acceleration data. Then, sensor data was aligned with gravity and transformed into the medical coordinate system. To detect initial contacts (commonly referred to as heel strikes) and estimate stride time, we applied the best-performing event detection algorithm from Seifer et al. [22]. Step length was subsequently estimated using a feature-based machine learning classifier (random forest), as introduced by Seifer et al. [23]. Finally, gait velocity was computed by combining stride time and step length [23]. All algorithms were previously validated against an optical motion capture system, which served as the gold standard, in the original publications [22,23].

#### 3.1.2. Foot-Worn Sensors

The Python package *gaitmap* [36] was used to process sensor data from the foot-worn IMUs. Similar to *EarGait*, *gaitmap* is an open-source code library for gait-related algorithms and IMU-related functions specifically developed for foot-worn sensors. First, individual strides were segmented using a dynamic time-warping algorithm, followed by manual inspection and correction. Sensor data was then aligned with gravity and transformed into the medical coordinate system. For gait event detection and gait parameter estimation, an end-to-end pipeline was applied based on the *Rampp* algorithm for event detection and temporal parameter estimation. This was followed by the *Madgwick* algorithm to obtain sensor orientation and the *ForwardBackwardIntegration* algorithm to obtain the position in world coordinates. Based on these, step length and gait velocity can be estimated for each step.

### 3.2. Statistics

Statistical differences were investigated using repeated-measures (RM) ANOVA for each gait parameter. The assumption of normality was assessed using the Shapiro–Wilk test. Greenhouse–Geisser corrections were applied if the assumption of sphericity, indicated by the Mauchly test, was violated. As post hoc tests, we used pairwise *t*-tests and applied Bonferroni corrections to counteract for multiple comparisons. The significance level α was set to 0.05, and effect sizes are reported as $n^{2}$ for the RM ANOVAs and Hedges’ *g* for *t*-tests. In all Tables, we use the following notation to indicate statistical significance: * *p* < 0.05, ** *p* < 0.01, *** *p* < 0.001. The open-source statistical package *pingouin* [37] was used for all statistical tests.

### 3.3. Evaluation

Word recognition performance (WRP) for the Freiburger monosyllabic word test was assessed in four different conditions: ON and OFF while sitting or walking, respectively. The statistical differences were assessed using RM ANOVA as described in the previous section, with HA status and task as within-subject factors. To assess gait parameters, we computed the average stride time, step length, and gait velocity for each walking bout. Statistical differences in gait parameters across conditions were analyzed using the ear-worn system and a 2 × 3 RM ANOVA, with HA status and tasks as within-subject factors.

The dual-task costs (DTCs) are estimated by:

(1) DTC=dual−single,

where single refers to the parameter from the single task (*Base_Walk_* or *Base_Speech_*) and dual refers to the same parameter from the corresponding dual task (*DT_Listen_* or *DT_Calc_*). DTCs were estimated for the walking parameters (DTC_Gait_) and listening test performance (DTC_Speech_).

The absolute error (AE) and absolute percentage error (APE) between the foot and ear measurement systems were estimated as follows:

(2) $$AE=|x_{foot}-x_{ear}|$$

(3) $$APE=|\frac{x_{foot}-x_{ear}}{x_{foot}}|\;\cdot \;100\%,$$

where *x* represents the specific gait parameter, i.e., gait velocity, step length, or stride time.

It should be noted that in this study, foot-worn sensors were employed solely as an additional reference system; while the ear-worn gait analysis system has demonstrated strong performance in previous validation studies [22,23], it remains a relatively novel measurement approach. To enhance the credibility of our findings, foot-worn sensors were incorporated as well-established and independent references. As described in detail in Section 4.3, the comparison revealed a strong correlation between the two systems. Consequently, all statistical analyses were conducted using gait parameters derived from the ear-worn system as it best supports the goal of unobtrusive mobility assessment via ubiquitous ear-worn sensors.

## 4. Results

This section presents the effect of HA amplification and dual-task conditions on both speech intelligibility and gait performance. Speech intelligibility was assessed using the Freiburger monosyllabic word test. The data of one participant were excluded from the statistical analysis of gait parameters because the participant was not able to complete the *DT_Calc_*. Additionally, gait parameter estimations from ear- and foot-worn sensors were compared.

### 4.1. Effects on Speech Intelligibility

**Figure 3:**
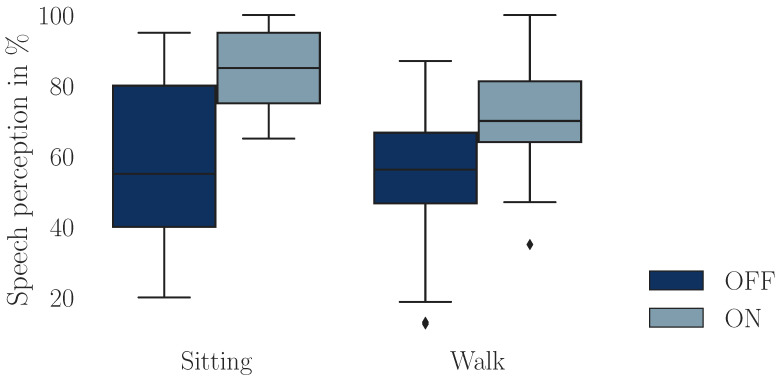
Boxplot illustrating speech perception performance in the Freiburger monosyllabic speech test in a sitting position and while walking. Both conditions were performed without (OFF) and with (ON) hearing aid amplification.

The analysis of recognition performance in the Freiburger monosyllabic word test revealed a notable improvement with HA amplification, regardless of whether participants were sitting or walking (Figure 3). As presented in Table 2, the WRP increased by about 23% in the sitting condition and by 17% during walking. Results from the RM ANOVA confirmed a highly significant main effect of HA amplification on speech perception (F(1, 24), p<0.001, $n^{2}=0.240$), demonstrating that HA amplification substantially enhances WRP. However, no significant effect of task condition or interaction between the task and the HA status was found. Dual-task costs for speech perception (DTC_Speech_) were higher in the ON condition (Table 3), aligning with the post hoc paired *t*-tests. A significant difference was found between *Base_Speech_* and *DT_Listen_* in the ON (t(24), p=0.029, g=0.714), whereas no significant difference was observed in the OFF condition.

### 4.2. Effects on Gait

The analysis of the DTC for gait parameters revealed a notable impact of dual-tasking on walking performance. As shown in Table 3, gait velocity and step length decreased under dual-task conditions, with reductions ranging from 0.041 to 0.075 m s^−1^ and 1.4 to 1.7 cm, respectively. In contrast, stride time showed positive DTCs, meaning that it increased in DT conditions. The DTCs for *Listen* were slightly less compared to *Calc*. Similarly, the DTCs for OFF were slightly lower compared to the ON condition.

The 2 × 3 RM ANOVA was conducted to examine the effects of HA amplification during the different tasks on gait parameters. The analysis revealed a significant main effect of the task on all three gait parameters (p<0.001, Table 4), indicating that task complexity influences walking performance. In contrast, hearing aid status did not have a significant main effect on any of the gait parameters, suggesting that gait characteristics remain largely unchanged between the ON and OFF conditions. A significant interaction between task type and HA status was found for stride time (F(2,46)=6.085, p=0.005, $n^{2}=0.004$), indicating that the impact of task complexity on stride time varied depending on whether the hearing aid was active. The post hoc analyses showed that gait velocity and step length were significantly lower in the *DT_Calc_* and *DT_Listen_* conditions compared to the *Base_Gait_* condition (p<0.001; Appendix A Table A2). For stride time, significant differences emerged between task conditions, particularly when the hearing aid was ON. Stride time was longest in the *DT_Calc_* condition and shortest in the *Base_Gait_* condition, with *DT_Listen_* falling in between. The influence of HA status on stride time was most pronounced in the *DT_Calc_* task, where the ON condition resulted in greater differences compared to the OFF condition. A comprehensive list of the absolute gait parameters for all dual tasks and hearing conditions is provided in the Appendix A (Table A1).

### 4.3. Ear vs. Foot Sensors

**Figure 4:**
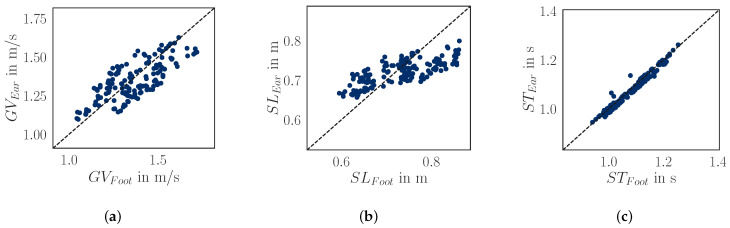
Scatter plot illustrating gait parameter estimations obtained from foot- and ear-worn sensors. Additionally, the absolute error (AE) and the absolute percentage error (APE) are reported (as mean ± standard deviation). Each data point represents the parameter (e.g., stride time) averaged over a walking bout of an individual participant. (**a**) Gait velocity; (**b**) step length; (**c**) stride time.

We compared gait parameters (gait velocity, step length, and stride time) obtained from foot-worn and ear-worn sensors. All parameters show a strong correlation (Table 5), with the strongest correlation for the stride time. The APE for stride time is 1% ± 0.8% (AE = 0.01 s ± 0.01 s). In contrast, the errors for step length and gait velocity estimations were slightly higher, with 6.4% ± 3.8% (AE = 0.047 m ± 0.030 m) and 6.6% ± 3.7% (AE = 0.09 m s^−1^ ± 0.05 m s^−1^), respectively. The correlation for gait speed estimations between the two sensor systems is illustrated in Figure 4.

## 5. Discussion

This study aimed to analyze the impact of hearing aid amplification on gait parameters in complex environments using a novel hearing-aid integrated gait analysis system. To the authors’ knowledge, this is the first study to examine the walking patterns of individuals with HL when walking aided and unaided during dual-task scenarios designed to mimic real-world cognitive load. While we did not observe a significant difference in gait parameters between the aided and unaided conditions, the DTCs were unexpectedly low. This suggests that the applied dual tasks may have failed to sufficiently increase cognitive load and replicate a truly complex environment. Consequently, our findings indicate that in simple environments, hearing aid amplification does not impact walking behavior. Importantly, this work is the first study to use the novel ear-worn gait analysis system for analyzing an audiology-specific research question. We demonstrate that it can estimate gait parameters with good precision and consistent with prior technical validation studies. These results highlight the reliability of the system and its potential for real-world, long-term home recordings to assess walking behavior in genuinely complex environments outside the laboratory.

### 5.1. DTCs—Always Check Your DTCs

In this study, we used a dual-task paradigm to evaluate the impact of HA amplification on walking behavior. DT paradigms are widely used to simulate real-world conditions with cognitive load while maintaining control in the laboratory setting. Contrary to our initial hypothesis, no significant difference was observed in gait velocity and step length between OFF and ON conditions, and only a small effect was found for stride time. These findings suggest that hearing aid amplification is unlikely to have a meaningful impact on walking behavior.

Additionally, we expected higher DTCs in the OFF condition, particularly in the *Listen* task, as costs typically increase with task complexity [38,39]. However, this was not the case, as DTCs for OFF were lower compared to the ON condition. Similarly, the inability to hear was expected to increase cognitive demand compared to the *DT_Calc_* task, given the *DT_Listen_* task’s direct reliance on hearing.

Although we observed significant gait differences between single and dual tasks, our absolute DTCs were substantially lower than those reported in the literature. Smith et al. [40] reported average DTCs of 0.18 m/s (95%CI: [0.15, 0.22]) across 25 studies, whereas we observed only 0.06 m/s; while the diversity of dual tasks in Smith et al.’s review makes direct comparison difficult due to the influence of task type [41,42], the work of Hausdorff et al. [43] employed the same arithmetic DT as this work. They report notably higher DTC of 0.15 m/s, approximately twice as high as in our study. These discrepancies suggest that our dual-task paradigm did not impose the expected cognitive demand.

A potential explanation is that participants may have prioritized gait over the cognitive task, leading to only minor changes in gait parameters. Given individual differences, some participants may have focused on maintaining walking stability at the expense of cognitive performance, leading to minimal DTCs for gait but substantial DTCs for cognition; while we cannot control individual task prioritization strategies, we can assess the (negative) correlations between DTCs in speech and gait. However, our analysis did not reveal such a correlation (Appendix A Figure A2), suggesting that, in our case, the differences in task prioritization do not account for our findings. Furthermore, in the OFF condition, there was no significant difference between speech intelligibility (Figure 3), which also indicates that participants were able to maintain their performance on the auditory task despite potential prioritizing gait. Beyond these observations, task prioritization has been shown to influence the magnitude of DTCs [44]; however, prioritizing gait reduces DTCs only slightly, with values around 0.13 m/s [45]. Given that our DTCs were considerably lower than expected, task prioritization alone is unlikely to explain the observed results.

Another plausible explanation is that our experimental setup imposed an inherent baseline cognitive demand for all conditions, potentially influenced by factors such as the novel environment or the specific acoustic conditions introduced by the HAs. We did not perform real-ear-aided-gain measurements to verify whether the prescribed amplification was achieved at the eardrum [46]. As a result, it is possible that some participants did not receive the full intended gain, potentially increasing cognitive listening effort even in the ON condition. However, the word recognition rate significantly improved by more than 20% (Figure 3) in the ON condition, showing that participants had a clear benefit. Moreover, an additional walking bout recorded with foot-worn sensors and the participant’s own HA revealed no significant difference in gait parameters when compared to the *Base ON* recording, suggesting that HA amplification functioned as intended.

The average PTA4 in this study was approximately 40 dB, indicating mild to moderate hearing loss, which may have influenced the effect size, as higher levels of HL have been associated with greater declines in gait parameters [11]. However, even mild HL has been associated with greater DTCs [11] than those observed in this study. Furthermore, a supplementary analysis revealed a negligible correlation between DTCs and HL severity, suggesting that the lower DTCs are not primarily driven by the degree of hearing loss.

Nevertheless, the unexpectedly low DTC prevented us from fully addressing our primary research question and underscored an important lesson: Always check your DTCs. Pilot testing or intermediate validation could have identified these issues earlier, highlighting the need for rigorous design and validation of dual-task paradigms. However, we did not deem this necessary due to the clear evidence that could be derived from our literature analysis. Future studies should ensure that dual-task demands are sufficient to align with the research objectives and produce realistic insights into real-world gait challenges.

In summary, previous studies consistently demonstrated a clear reduction in gait performance under cognitively demanding conditions, regardless of task prioritization. Since such reductions were not observed in our study, we conclude that the applied dual-task paradigm did not induce the expected cognitive load. Consequently, no definitive conclusions can be drawn regarding the effects of HA amplification on gait in cognitively demanding situations. However, our findings still provide valuable insights into walking behavior and the role of HA amplification in low-cognitive-load environments, serving as a foundation for future investigations under more ecologically valid conditions.

### 5.2. Negligible Impact of HA in Simple Environments

Although our findings on DTCs do not allow conclusions about complex environments, they do provide insights into gait behavior under simpler walking conditions. Across the three different walking tasks (*Base_Gait_, DT_Listen_, DT_Calc_*), we observed significant variation in gait parameters but no discernible impact of HA amplification. Significant differences between tasks were expected, as dual-task paradigms are designed to impose varying cognitive demands and assess their impact on performance [43]. The absence of significant differences between the ON and OFF conditions suggests that individuals with HL can perform basic walking tasks without any apparent gait impairments, even when unaided. A significant interaction effect between HA status and task conditions was observed for stride time, indicating that the influence of task demands on stride time varies depending on whether the hearing aid is ON or OFF. Post hoc analyses revealed that, with the exception of one comparison, most effects were small, suggesting that dual-task conditions exert a measurable but limited impact on stride time. In contrast, no significant interaction effects were found for step length or gait velocity. Existing literature suggests that individual gait parameters are commonly affected to varying degrees by dual-tasking [41,45]. However, given the relatively small effect size observed for stride time and the absence of significant interactions for other gait parameters, the overall impact of HA status on gait across different walking conditions appears to be negligible.

Note that statistical analyses were based on the ear-worn system. A supplementary RM ANOVA using gait parameters derived from foot-worn sensors yielded the same statistical outcomes (see Appendix A Table A3).

Our findings regarding the impact of HA amplification align with those of Weaver et al. [47], who reported no significant changes in gait walking aided and unaided, though their recordings lacked realism as participants were walking blindfolded. Similarly, the study by Goodwin et al. [15], which analyzed different health outcomes between a baseline measurement and a six-week follow-up in individuals with HAs, did not find a statistically significant difference between the measures. However, since the study primarily aimed to assess the feasibility of the study protocol rather than to detect measurable changes within a short time frame, the absence of significant differences was not unexpected. It is important to emphasize that our results do not imply that HL has no effect on gait, as it has been reported by many others [8,9,11,48]. Rather, our findings suggest that HA amplification does not induce additional gait alterations.

Overall, our study highlights the resilience of participants’ motor performance in individuals with HL when cognitive and environmental demands are minimal. However, as this was only a pilot study with 25 participants, further research is needed to investigate and understand the underlying mechanism of hearing loss-related gait changes in complex and cognitively demanding environments. The small sample size limits the statistical power and generalizability of our results. A larger, more diverse participant pool is necessary to validate our conclusions and ensure their applicability to a broader population. Future studies should include more participants to strengthen the reliability of our findings on the impact of hearing aid amplification on gait in low cognitive demanding environments.

### 5.3. Individuals Showing Benefits

**Figure A1:**
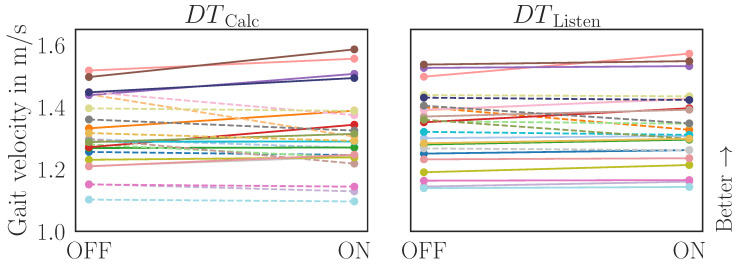
Slope graphs of gait velocity for the dual tasks Calc and Listen. Each line represents a single participant. Solid lines represent increasing gait velocity for the ON condition; dashed lines represent decreasing gait velocity.

**Figure A2:**
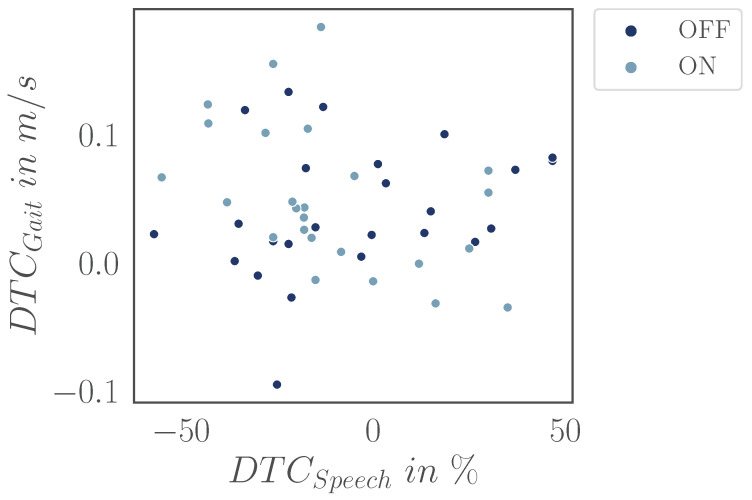
Scatter plot of dual-task costs (DTCs) for gait (*DTC_Gait_*) and speech intelligibility (*DTC_Speech_*). The data suggest a weak to moderate correlation for the ON and OFF conditions (Pearson correlation, $r_{ON}=-0.46$, $r_{OFF}=0.27$).

The analysis of individual gait changes revealed high variability across the participants (see Appendix A, Figure A1), with some showing improvements in gait parameters from HA amplification. This shows that the effects of HA on gait are not uniform among the participants. These findings align with previous research by Shayman et al. [49], who reported that hearing interventions may lead to significant gait improvements, although these effects are not uniform among patients. Notably, the work of Shayman et al. only included three participants and focused solely on baseline walking tasks without additional tasks. Similarly, Weaver et al. [47] found clinically relevant gait improvements when walking blindfolded in only a small subset of their study population.

### 5.4. Need for Standardized Acoustic DT

As discussed in Section 5.1, the DTCs for the arithmetic task were notably lower than those reported in the literature, suggesting that the applied cognitive demand was insufficient. Since the DTCs for the listening task were in a similar range, the same conclusion likely applies to the acoustic task as well. While it remains unclear why the standardized arithmetic DT in this study failed to produce the expected DTC, the results for the acoustic DT can not be compared to prior studies due to the lack of a standardized auditory dual-task paradigm. We selected a monosyllabic word test as alternative approaches were unsuitable for our study design. For instance, Lau et al. [50] used virtual reality environments and a treadmill, however treadmills walking has been shown to differ significantly from over-ground walking [51,52]. Others deployed a dichotic listening task requiring a multi-loudspeaker setup [53], impractical for long walking distances. To enable meaningful cross-study comparisons, a standardized acoustic dual-task paradigm is essential. Our results suggest that monosyllabic speech tests, such as the Freiburger, may not impose sufficient cognitive load to induce measurable DTC. A comprehensive evaluation is needed to establish an effective acoustic dual task involving testing across languages, diverse populations, and larger sample sizes. Developing a standardized framework would enhance reproducibility, facilitate comparisons across studies, and improve our understanding of cognitive load effects on walking in real-world scenarios.

### 5.5. Reliable Accuracy of Gait Algorithms for Ear-Worn Devices

Our algorithms demonstrated strong potential for gait analysis using ear-worn accelerometers with comparable performance to foot-worn sensors. This is particularly relevant as *EarGait* was applied for the first time to address a specific audiological research question related to gait, including generalizability and data quality.

The ear-specific gait algorithms used in this work were previously technically validated in controlled settings using an optical motion capture system, the gold standard for gait analysis [22,23]. An accuracy of 12 ms for stride time, 4.8 cm for step length, and 5.3% for gait velocity was reported. To further ensure measurement reliability, we integrated foot-worn sensors as a reference. While foot-worn IMUs are considered the most accurate for IMU-based gait analysis due to their direct coupling with foot motion, ear-worn sensors are more susceptible to upper body movements, which can introduce motion artifacts. However, the error between foot- and ear-derived estimates remained within the expected range of prior validation studies [22,23], reinforcing the robustness of our approach despite these inherent limitations.

The errors for step length and gait velocity were slightly higher compared to stride time, and no statistically significant effects were observed for these parameters. To ensure that these results were not confounded by potential inaccuracies of the ear-worn system, we performed an additional RM ANOVA using gait parameters derived from foot-worn sensors. The statistical outcomes in terms of main and interaction effects were identical to those obtained with ear-worn data. This confirms that the minor differences between the measurement systems do not influence the overall findings regarding the effects of hearing aid amplification on gait.

The high accuracy of our analysis highlights the potential of ear-worn wearable (earable) devices for continuous mobility assessments in real-world environments. Our comparison with foot-worn sensors confirmed that changes in gait can be effectively measured using ear-worn IMUs. Although we did not observe a significant effect of HA amplification on gait—likely due to low DT demands—our secondary measurement system confirmed the accuracy of our gait analysis system. This suggests that our algorithms could reliably detect cognitive load-induced gait changes under more complex, real-life conditions. However, future studies are necessary to prove that.

Building on this potential, a hearing aid with an integrated accelerometer could serve not only as an auditory device but also as a mobility assessment tool, enabling the detection of gait deficits in individuals with hearing impairments. In the context of Meniere’s disease—a disorder of the inner ear characterized by episodes of vertigo, hearing loss, and tinnitus—continuous monitoring of mobility could provide valuable clinical insights, particularly given the disease’s impact on balance and gait stability. Furthermore, this approach could also be extended to cochlear implants, as users often experience balance and gait impairments that may improve following implantation [54,55]. However, current limitations in processing power and battery life pose challenges for real-time analysis. Future advancements in hardware and algorithm optimization could enable continuous gait monitoring, aiding in fall risk detection and adaptive auditory interventions based on cognitive demands. An integrated accelerometer could enable unobtrusive monitoring of mobility and support rehabilitation tracking after implantation. Beyond hearing devices, our approach could extend to other ear-worn devices, like earbuds, for broader healthcare monitoring and telemedicine applications.

## 6. Conclusions

This study examined the effect of HA amplification on gait parameters using ear-worn accelerometers. Our findings showed no significant impact of HA on gait in various controlled, low-cognitive-demand environments. However, the ear-worn gait analysis system showed high accuracy, highlighting its potential for real-world mobility monitoring. Future research should explore more complex real-world scenarios to deepen our understanding of the relationship between hearing loss, cognitive load, and gait stability. Ear-worn sensors offer a promising tool for unobtrusive and continuous mobility assessment in real-world scenarios.

## Figures and Tables

**Table 2:** Mean (±standard deviation) word recognition performance (WRP) for the Freiburger monosyllabic word test. The test was conducted in sitting and walking conditions, both with (ON) and without (OFF) hearing aid amplification. The results of the repeated-measures (RM) ANOVA are also reported, showing the effect of task, hearing aid status, and their interaction. Significance levels are indicated as follows: *** *p* < 0.001.

WRP in % Task	OFF	ON	
Sitting	61.0 ± 21.8	84.2 ± 12.2	
Walking	54.2 ± 21.5	71.2 ± 17.5	
**RM ANOVA Measure**	**F(1, 24)**	***p***	$n^{2}$
Task	2.876	0.103	0.043
HA status	43.570	<0.001 ***	0.240
Task:HA status	1.972	0.173	0.008

**Table 3:** The dual-task costs for the gait (DTC_Gait_) and speech intelligibility tasks (DTC_Speech_). Gait parameters were estimated when walking without (OFF) and with (ON) hearing aid (HA) amplification. Word recognition performance (WRP) was assessed while sitting and walking, with and without hearing aid amplification, using the Freiburger monosyllabic word test.

		DTC_Gait_			DTC_Speech_
**Dual Task**	**HA Status**	**Gait Velocity in m/s**	**Step Length in m**	**Stride Time in s**	**WRP in %**
*DT_Calc_*	OFF	−0.061	−0.017	0.022	
	ON	−0.075	−0.015	0.042	
*DT_Listen_*	OFF	−0.040	−0.016	0.003	−4.7
	ON	−0.049	−0.014	0.018	−11.2

**Table 4:** Repeated-measures ANOVA results for the gait parameters. The table presents the main effects of task and hearing aid (HA) status, as well as the interaction effect between task and HA status. Significance levels are indicated as follows: ** *p* < 0.01, *** *p* < 0.001.

	Gait Velocity			Step Length			Stride Time		
**Measure**	**F(2, 46)**	***p***	$n^{2}$	**F(2, 46)**	***p***	$n^{2}$	**F(2, 46)**	***p***	$n^{2}$
Task	26.744	<0.001 ***	0.055	21.551	<0.001 ***	0.064	22.200	<0.001 ***	0.035
HA status	1.490	0.235	0.001	2.368	0.137	0.001	0.723	0.404	0.000
Task:HA status	0.727	0.468	0.001	0.138	0.845	0.000	6.085	0.005 **	0.004

**Table 5:** Absolute error (AE), absolute percentage error (APE), and Pearson correlation coefficient (*r*) for different gait parameters measured using foot- and ear-worn sensors. Values are presented as the mean ± standard deviation.

	AE	APE	Pearson
	**Mean ± Std**	**Mean ± Std**	**Correlation** ***r***
Gait velocity	0.09±0.05 m/s	6.6±3.7%	0.75
Step length	0.047±0.030 m	6.4±3.8%	0.74
Stride time	0.01±0.01 s	1.0±0.8%	0.99

## Data Availability

No data from this study are publicly available. The participants of the present study did not consent to the publication of their data in open repositories, in accordance with European data protection laws.

## Abbreviations

The following abbreviations are used in this manuscript:

AE	Absolute error
APE	Absolute percentage error
DT	Dual-task
DTC	Dual-task cost
HA	Hearing aid
HL	Hearing loss
IMU	Inertial measurement unit
RM ANOVA	Repeated-measures ANOVA
STD	Standard deviation
WRP	Word recognition performance

## Appendix A

### Appendix A.1

**Table A1:** Mean and standard deviation (std) of gait parameters, including gait velocity, step length, and stride time, for the different scenarios. The scenarios include single-task walking (Base), the dual task of performing a calculation while walking (Calc), and the dual task of performing a word recognition listening test while walking (Listen). Data are presented for all conditions combined (All) and separately for the unaided (OFF) and aided (ON) conditions.

		Gait Velocity in m/s		Step Length in m		Stride Time in s	
	**HA Status**	**Mean**	**Std**	**Mean**	**Std**	**Mean**	**Std**
All	All	1.343	0.120	0.720	0.029	1.082	0.072
	OFF	1.340	0.117	0.719	0.028	1.083	0.070
	ON	1.346	0.125	0.721	0.030	1.080	0.074
Base_Gait_	All	1.380	0.120	0.730	0.029	1.067	0.068
	OFF	1.374	0.120	0.730	0.029	1.074	0.070
	ON	1.387	0.122	0.731	0.030	1.060	0.068
DT_Calc_	All	1.312	0.118	0.714	0.028	1.099	0.077
	OFF	1.312	0.112	0.713	0.025	1.096	0.074
	ON	1.312	0.127	0.715	0.030	1.102	0.081
DT_Listen_	All	1.336	0.114	0.715	0.029	1.078	0.068
	OFF	1.334	0.114	0.714	0.029	1.078	0.068
	ON	1.338	0.118	0.717	0.029	1.078	0.069

**Table A2:** Post hoc analysis for gait parameters (paired *t*-tests) with the Bonferroni correction. Due to the significant interaction between hearing aid (HA) status and tasks for stride time, a separate post hoc analysis was performed for each HA status (OFF/ON). Significance levels are indicated as follows: * *p* < 0.05, ** *p* < 0.01, *** *p* < 0.001.

		Gait Velocity			Step Length		
		**t(23)**	***p***	**Hedges’ g**	**t(23)**	***p***	**Hedges’ g**
Base_Gait_	DT_Calc_	6.445	<0.001 ***	0.565	5.423	<0.001 ***	0.567
	DT_Listen_	4.409	0.001 **	0.369	5.353	<0.001 ***	0.512
DT_Calc_	DT_Listen_	−3.209	0.012 *	−0.204	−0.466	>0.99	−0.040
		**Stride Time**					
		**OFF**			**ON**		
		**t(23)**	***p***	**Hedges’ g**	**t(23)**	***p***	**Hedges’ g**
Base_Gait_	DT_Calc_	−3.304	0.019 *	−0.303	−6.568	<0.001 ***	−0.554
	DT_Listen_	−0.563	>0.999	−0.048	−4.212	0.002 **	−0.255
DT_Calc_	DT_Listen_	4.984	<0.001 ***	0.259	3.656	0.008 **	0.319

**Table A3:** Repeated-measures ANOVA results for the gait parameters estimated with foot-worn sensors. The table presents the main effects of task and hearing aid (HA) status, as well as the interaction effect between task and HA status. Significance levels are indicated as follows: * *p* < 0.05, *** *p* < 0.001. Statistical outcomes align with statistical analysis based on ear-worn sensors.

	Gait Velocity			Step Length			Stride Time		
**Measure**	**F(2, 46)**	***p***	$n^{2}$	**F(2, 46)**	***p***	$n^{2}$	**F(2, 46)**	***p***	$n^{2}$
Task	32.962	<0.001 ***	0.040	22.246	<0.001 ***	0.017	30.259	<0.001 ***	0.037
HA status	0.767	0.390	0.000	1.214	0.282	0.000	0.098	0.757	0.000
Task: HA status	0.974	0.380	0.00	0.626	0.530	0.000	4.361	0.020 *	0.001

## References

[1] World Health Organization—WHO (2024). Deafness and Hearing Loss. https://www.who.int/news-room/fact-sheets/detail/deafness-and-hearing-loss.

[2] Lawrence B.J., Jayakody D.M.P., Bennett R.J., Eikelboom R.H., Gasson N., Friedland P.L. (2020). Hearing Loss and Depression in Older Adults: A Systematic Review and Meta-analysis. Gerontologist.

[3] Lin F.R., Yaffe K., Xia J., Xue Q.L., Harris T.B., Purchase-Helzner E., Satterfield S., Ayonayon H.N., Ferrucci L., Simonsick E.M. (2013). Hearing loss and cognitive decline in older adults. JAMA Intern. Med..

[4] Agmon M., Lavie L., Doumas M. (2017). The association between hearing loss, postural control, and mobility in older adults: A systematic review. J. Am. Acad. Audiol..

[5] Besser J., Stropahl M., Urry E., Launer S. (2018). Comorbidities of hearing loss and the implications of multimorbidity for audiological care. Hear. Res..

[6] Martinez-Amezcua P., Powell D., Kuo P.L., Reed N.S., Sullivan K.J., Palta P., Szklo M., Sharrett R., Schrack J.A., Lin F.R. (2021). Association of age-related hearing impairment with physical functioning among community-dwelling older adults in the US. JAMA Netw. Open.

[7] Foster J.I., Williams K.L., Timmer B.H.B., Brauer S.G. (2022). The association between hearing impairment and postural stability in older adults: A systematic review and meta-analysis. Trends Hear..

[8] Viljanen A., Kaprio J., Pyykkö I., Sorri M., Pajala S., Kauppinen M., Koskenvuo M., Rantanen T. (2009). Hearing as a predictor of falls and postural balance in older female twins. J. Gerontol. Ser. Biol. Sci. Med Sci..

[9] Chen D.S., Betz J., Yaffe K., Ayonayon H.N., Kritchevsky S., Martin K.R., Harris T.B., Purchase-Helzner E., Satterfield S., Xue Q.L. (2015). Association of hearing impairment with declines in physical functioning and the risk of disability in older adults. J. Gerontol. Ser. Biol. Sci. Med Sci..

[10] Bessot N., Denise P., Toupet M., Van Nechel C., Chavoix C. (2012). Interference between walking and a cognitive task is increased in patients with bilateral vestibular loss. Gait Posture.

[11] Wollesen B., Scrivener K., Soles K., Billy Y., Leung A., Martin F., Iconomou N., McMahon C., Dean C. (2018). Dual-task walking performance in older persons with hearing impairment: Implications for interventions from a preliminary observational study. Ear Hear..

[12] Viljanen A., Kaprio J., Pyykkö I., Sorri M., Koskenvuo M., Rantanen T. (2009). Hearing acuity as a predictor of walking difficulties in older women. J. Am. Geriatr. Soc..

[13] Wingfield A., Tun P.A., McCoy S.L. (2005). Hearing loss in older adulthood: What it is and how it interacts with cognitive performance. Curr. Dir. Psychol. Sci..

[14] Campos J., Ramkhalawansingh R., Pichora-Fuller M.K. (2018). Hearing, self-motion perception, mobility, and aging. Hear. Res..

[15] Goodwin M.V., Slade K., Kingsnorth A.P., Urry E., Maidment D.W. (2025). Can hearing aids improve physical activity in adults with hearing loss? A feasibility study. Audiol. Res..

[16] Martinez-Amezcua P., Kuo P.L., Reed N.S., Simonsick E.M., Agrawal Y., Lin F.R., Deal J.A., Ferrucci L., Schrack J.A. (2021). Association of hearing impairment with higher-level physical functioning and walking endurance: Results from the Baltimore Longitudinal Study of Aging. J. Gerontol. Ser. A.

[17] Mahafza M.T., Wilson W.J., Brauer S., Timmer B.H.B., Hickson L. (2022). A systematic review of the effect of hearing aids on static and dynamic balance in adults with hearing impairment. Trends Hear..

[18] Borsetto D., Corazzi V., Franchella S., Bianchini C., Pelucchi S., Obholzer R., Soulby A.J., Amin N., Ciorba A. (2021). The influence of hearing aids on balance control: A systematic review. Audiol. Neurotol..

[19] Röddiger T., Clarke C., Breitling P., Schneegans T., Zhao H., Gellersen H., Beigl M. (2022). Sensing with earables: A systematic literature review and taxonomy of phenomena. Proc. ACM Interact. Mob. Wearable Ubiquitous Technol..

[20] Diao Y., Ma Y., Xu D., Chen W., Wang Y. (2020). A novel gait parameter estimation method for healthy adults and postoperative patients with an ear-worn sensor. Physiol. Meas..

[21] Decker J., Boborzi L., Schniepp R., Jahn K., Wuehr M. (2024). Mobile spatiotemporal gait segmentation using an ear-worn motion sensor and deep learning. Sensors.

[22] Seifer A.K., Dorschky E., Küderle A., Moradi H., Hannemann R., Eskofier B.M. (2023). EarGait: Estimation of temporal gait parameters from hearing aid integrated inertial sensors. Sensors.

[23] Seifer A.K., Küderle A., Dorschky E., Moradi H., Hannemann R., Eskofier B.M. (2024). Step length and gait speed estimation using a hearing aid integrated accelerometer: A comparison of different algorithms. IEEE J. Biomed. Health Inform..

[24] Freepik Designed by Stories Freepik. www.freepik.com.

[25] Nasreddine Z.S., Phillips N.A., Bédirian V., Charbonneau S., Whitehead V., Collin I., Cummings J.L., Chertkow H. (2005). The Montreal Cognitive Assessment, MoCA: A brief screening tool for mild cognitive impairment. J. Am. Geriatr. Soc..

[26] Dias N., Kempen G., Todd C., Beyer N., Freiberger E., Piot-Ziegler C., Yardley L., Hauer K. (2006). The German version of the falls efficacy scale-international version (FES-I). Z. Gerontol. Geriatr..

[27] Podsiadlo D., Richardson S. (1991). The timed “Up & Go”: A test of basic functional mobility for frail elderly persons. J. Am. Geriatr. Soc..

[28] Pavasini R., Guralnik J., Brown J.C., di Bari M., Cesari M., Landi F., Vaes B., Legrand D., Verghese J., Wang C. (2016). Short physical performance battery and all-cause mortality: Systematic review and meta-analysis. BMC Med..

[29] Keidser G., Dillon H., Carter L., O’Brien A. (2012). NAL-NL2 empirical adjustments. Trends Amplif..

[30] Hahlbrock K.H. (1953). Über Sprachaudiometrie und neue Wörterteste. Arch. Ohren-Nasen-und Kehlkopfheilkd..

[31] Amboni M., Barone P., Hausdorff J.M. (2013). Cognitive contributions to gait and falls: Evidence and implications. Mov. Disord..

[32] Shumway-Cook A., Brauer S., Woollacott M. (2000). Predicting the probability for falls in community-dwelling older adults using the Timed Up & Go test. Phys. Ther..

[33] Dalrymple-Alford J., MacAskill M., Nakas C., Livingston L., Graham C., Crucian G., Melzer T., Kirwan J., Keenan R., Wells S. (2010). The MoCA: Well-suited screen for cognitive impairment in Parkinson disease. Neurology.

[34] Delbaere K., Close J.C.T., Mikolaizak A.S., Sachdev P.S., Brodaty H., Lord S.R. (2010). The Falls Efficacy Scale International (FES-I). A comprehensive longitudinal validation study. Age Ageing.

[35] Seifer A.K., Küderle A. EarGait: A Gait Analysis Package for Ear-Worn IMU Sensors. (GitHub, Version 2.11.0). 2022. https://github.com/mad-lab-fau/eargait.

[36] Küderle A., Ullrich M., Roth N., Ollenschläger M., Ibrahim A.A., Moradi H., Richer R., Seifer A.K., Zürl M., Sîmpetru R.C. (2024). Gaitmap—An open ecosystem for IMU-based human gait analysis and algorithm benchmarking. IEEE Open J. Eng. Med. Biol..

[37] Pingouin: Pingouin Is an Open-Source Statistical Package Written in Python. Version 0.5.4. https://pingouin-stats.org.

[38] Lövdén M., Schaefer S., Pohlmeyer A.E., Lindenberger U. (2008). Walking variability and working-memory load in aging: A dual-process account relating cognitive control to motor control performance. J. Gerontol..

[39] Montero-Odasso M., Muir S.W., Speechley M. (2012). Dual-task complexity affects gait in people with mild cognitive impairment: The interplay between gait variability, dual tasking, and risk of falls. Arch. Phys. Med. Rehabil..

[40] Smith E., Cusack T., Blake C. (2016). The effect of a dual task on gait speed in community dwelling older adults: A systematic review and meta-analysis. Gait Posture.

[41] Beurskens R. (2013). Does the walking task matter? Influence of different walking conditions on dual-task performances in young and older persons. Hum. Mov. Sci..

[42] Beauchet O., Dubost V., Aminian K., Gonthier R., Kressig R.W. (2005). Dual-task-related gait changes in the elderly: Does the type of cognitive task matter?. J. Mot. Behav..

[43] Hausdorff J.M., Schweiger A., Herman T., Yogev-Seligmann G., Giladi N. (2008). Dual-task decrements in gait: Contributing factors among healthy older adults. J. Gerontol. Ser. Biol. Sci. Med Sci..

[44] Lee J., Park S. (2018). Effects of a priority-based dual task on gait velocity and variability in older adults with mild cognitive impairment. J. Exerc. Rehabil..

[45] Yogev-Seligmann G., Rotem-Galili Y., Mirelman A., Dickstein R., Giladi N., Hausdorff J.M. (2010). How does explicit prioritization alter walking during dual-task performance? Effects of age and sex on gait speed and variability. Phys. Ther..

[46] Alberti G., Portelli D., Loteta S., Galletti C., D’Angelo M., Ciodaro F. (2024). Open-fitting hearing aids: A comparative analysis between open behind-the-ear and open completely-in-the-canal instant-fit devices. Eur. Arch. Oto-Rhino-Laryngol..

[47] Weaver T., Shayman C., Hullar T. (2017). The effect of hearing aids and cochlear implants on balance during gait. Otol. Neurotol..

[48] Cornwell T., Woodward J., Wu M., Jackson B., Souza P., Siegel J., Dhar S., Gordon K.E. (2020). Walking with ears: Altered auditory feedback impacts gait step length in older adults. Front. Sport. Act. Living.

[49] Shayman C.S., Earhart G.M., Hullar T.E. (2017). Improvements in gait with hearing aids and cochlear implants. Otol. Neurotol..

[50] Lau S.T., Pichora-Fuller M.K., Li K.Z.H., Singh G., Campos J.L. (2016). Effects of hearing loss on dual-task performance in an audiovisual virtual reality simulation of listening while walking. J. Am. Acad. Audiol..

[51] Hollman J.H., Watkins M.K., Imhoff A.C., Braun C.E., Akervik K.A., Ness D.K. (2016). A comparison of variability in spatiotemporal gait parameters between treadmill and overground walking conditions. Gait Posture.

[52] Lazzarini B., Kataras T.J. (2016). Treadmill walking is not equivalent to overground walking for the study of walking smoothness and rhythmicity in older adults. Gait Posture.

[53] Gorecka M.M., Vasylenko O., Rodríguez-Aranda C. (2020). Dichotic listening while walking: A dual-task paradigm examining gait asymmetries in healthy older and younger adults. J. Clin. Exp. Neuropsychol..

[54] Murray D., Viani L., Garvan J., Murphy A., Vance R., Simoes-Franklin C., Smith J., Meldrum D. (2020). Balance, gait and dizziness in adult cochlear implant users: A cross sectional study. Cochlear Implant. Int..

[55] Kaczmarczyk K., Błażkiewicz M., Wiszomirska I., Pietrasik K., Zdrodowska A., Wit A., Barton G., Skarżyński H. (2019). Assessing Gait Stability before and after Cochlear Implantation. BioMed Res. Int..

